# Challenges in Applying Multimodal Imaging Technologies to Quantify In Vivo Glycogen and Intramuscular Fat in Livestock

**DOI:** 10.3390/foods14050784

**Published:** 2025-02-25

**Authors:** Tharcilla I. R. C. Alvarenga, Peter McGilchrist, Marianne D. Keller, David W. Pethick

**Affiliations:** 1 Armidale Livestock Industries Centre, NSW Department of Primary Industries and Regional Development, Armidale, NSW 2351, Australia; 2 School of Environmental and Rural Science, University of New England, Armidale, NSW 2351, Australia; peter.mcgilchrist@une.edu.au; 3 Sydney School of Veterinary Science, University of Sydney, Camperdown, NSW 2050, Australia; marianne.keller@sydney.edu.au; 4 South Australian Health and Medical Research Institute (SAHMRI), Adelaide, SA 5000, Australia; 5 Food Futures Institute, Murdoch University, Murdoch, WA 6150, Australia; d.pethick@murdoch.edu.au

**Keywords:** glycogen concentration, intramuscular fat, live animal, muscle hydration, ultrasound frequency

## Abstract

Predicting meat quality, especially dark, firm and dry meat, as well as muscle fat prior to slaughter, presents a challenge in practice. Medical as well as high-frequency ultrasound applications can be utilized to predict body composition and meat quality aspects. Ultrasounds are non-invasive, rapid-to-operate in vivo and show high correlations to the animal production traits being estimated. Farm animal ultrasounds are used to predict intramuscular fat content in the beef cattle industry. Challenges are identified in applying ultrasound technology to detect glycogen content in farm animals due to a wide range of fat, muscle and water composition. Other technologies and methods are reported in this literature review to overcome issues in the practicability and accuracy of ultrasound technology when estimating muscle glycogen levels in cattle. The discussion of other tools such as hyperspectral imaging, microwave sensor technology and digital infrared thermal imaging were addressed because of their superior accuracy in estimating moisture and fat components.

## 1. Introduction

Dark cutting meat, also referred to as dark, firm and dry meat (DFD) or high pH meat, is impacting beef quality in almost 10% of the carcasses in Australia, which means there is an almost $38 million loss per year [1]. From a farm-to-table perspective, muscle glycogen levels and the potential for DFD significantly impact the safety, shelf life and nutritional value of red meat. Stress at the farm level, leading to glycogen depletion, can result in darker, tougher meat that spoils more quickly and potentially loses nutritional quality [2]. Early detection of low muscle glycogen, the root cause of dark cutting meat, could help eliminate dark cutters prior to slaughter. Intramuscular fat content or marbling are important parameters used for genetic improvement, especially of beef cattle. Marbling is an important parameter related to meat tenderness, juiciness and flavor, with a reported correlation from 0.70 to 0.90 [3].

Methodologies have been studied to predict dark cutting beef and intramuscular fat content. Live animal characteristics and carcass traits have been studied as markers to predict dark cutting beef [4]. However, the correlation between the dark cutting and phenotype traits is weak given the large environmental influences on muscle glycogen metabolism.

Genetic parameters have indicated the possibility of identifying cattle with a predisposition to dark cutting [5,6]; however, the heritability of muscle glycogen level is moderate (h^2^ = 0.34) and the genetic correlation with carcass measurements is low [5]. In addition, the main cause of dark cutting has been associated with environmental factors like poor pre-slaughter nutrition combined with physical and emotional stress during transportation and other pre-slaughter management [7].

Although the changes in body water content challenge the prediction of muscle glycogen [8], it is known that ultrasound technology can be applied in athletes to assess and quantify muscle glycogen content [9]. To date, the use of ultrasound to quantify muscle glycogen on farm animals has not been investigated. The prediction of intramuscular fat content is moderately accurate when using ultrasound in live animals [10] and has been extensively investigated [10,11,12] given it is an economical way to contribute to genetic improvements and prediction of endpoints in production traits; however, it can be overestimated as fatness decreases or underestimated as fatness increases [13]. The potential use of ultrasound to determine muscle glycogen and intramuscular fat content in meat-producing animals has far-reaching implications from farm to table. At each stage of the food chain, this technology can provide data that improve meat quality, safety, shelf life and nutritional value. This literature and methodology review will address the potential use of ultrasound to determine muscle glycogen and intramuscular fat content of meat-producing animals.

## 2. Ultrasound Principles

Medical ultrasounds are based on the use of high-frequency sounds to aid in the diagnosis of patients. Ultrasound waves are transmitted into the body, usually in very short pulses. The waves are reflected at the surfaces between the tissues of different densities, the reflection being proportional to the difference in impedance. The returning echoes are analyzed to create an image. Usually, medical ultrasound frequencies are up to 20 MHz with an f-number of 0.5 (the ratio of the imaging depth to the aperture size). A medical treatment ultrasound with cauterization employs around 100 to 1000 MHz.

Higher MHz increases axial resolution and lateral resolution (20 and 100 μm), and an f-number of 2.9. However, higher frequency waves show more attenuation, meaning loss of wave amplitude (or “signal”) due to all mechanisms, including absorption, scattering and mode conversion [14], as the axial resolution increases.

## 3. Overview of Clinical and Farm Animal Applications of Ultrasound

### 3.1. Veterinary Companion Animal Ultrasound Applications

Ultrasound technology is used for pregnancy evaluations in animals, mostly in dogs, and is also used widely for diagnostics, including but not limited to eye diseases, urinary, tendons and muscle issues [15]. As such, ultrasounds are also used in horses to assess reproductive traits and healthiness of kidneys, stomach, duodenum and the transrectal of the large intestines [16]. On farms, transrectal ultrasounds have been used in bovine pregnancy evaluations [17].

High-frequency ultrasound scanners, which are designed to diagnose muscle glycogen in athletes, are currently used in the veterinary field to detect ligament damage and muscle ruptures, aiding the assessment of targeted canine rehabilitation [18].

### 3.2. Farm Animal Ultrasound Application, Including Meat Quality

Real-time ultrasound technology has been widely applied in several agricultural contexts for many years. In meat-producing animals, it has been used to improve meat texture [19], microbiological safety [20], shelf life and many other functional properties of meat products [21].

On farms, real-time ultrasound scanning has provided many practical solutions for live animal assessments such as pregnancy diagnosis, fertility and the prediction of carcass composition (Table 1). Rump fat depth, rib fat depth, eye muscle area and intramuscular fat are the most common attributes measured by ultrasound scanning to assess the carcass merit of an individual animal whilst still alive [22].

Ultrasound is a desirable tool to be applied on farms because it is a non-destructive method, rapid-to-operate in vivo and shows high correlations to the animal production traits being estimated. However, there are factors influencing the accuracy of ultrasonic measurements within and between species such as hide thickness, wool/hair, gender, body weight and body fat content.

In general, shearing or clipping the hair/wool to reduce its effect in the ultrasound image is important. In some studies, this effect is reduced by combining the hair to clean the skin surface [23] or applying vegetable oil as a coupling agent to improve the acoustic contact surface between the ultrasound probe and the skin [24].

Ultrasound measurements in sheep are influenced by the wool layer and the soft and loose outer layer of subcutaneous fat [25]. In pigs, it has been reported that the relative proportion of skin and backfat thickness affects the ultrasound velocity [26].

The thickness of goats’ subcutaneous fat changed from in vivo when compared to carcass post-mortem measurements [23]. For example, the sternum region showed a better prediction of fat depth (r = 0.92) using live animal ultrasound measurements when compared to the lumbar region (r = 0.63) due to the accessibility of subcutaneous fat in this region of goats. (Ultrasound measurement of sternum and lumbar fat depth: 29.34 mm and 3.62 mm, respectively. Carcass measurement of the sternum and lumbar fat depth: 25.19 mm and 1.14 mm, respectively [23]).

Ultrasound scanning closer to slaughter has greater accuracy to predict carcass traits than at the start of the finishing period [24,27].

**Table 1:** Ultrasound application on farms in monitoring carcass composition and reproductive physiology.

Parameter	Measurement Time	Location	Ultrasound Configuration	Specie	Reference
Carcass composition					
Loin area, subcutaneous fat thickness, marbling, rump fat thickness and gluteus medium depth.	Scanned at 30 ± 5 days on feed (approximately 250 days old), and then serially every 30 to 35 days throughout the finishing phase.	Between the 12th and 13th rib. At the junction of the biceps femoris and gluteus medius (GM) muscles between the ischium and illium.	Aquila Pro (Esaote Pie Medical) diagnostic real-time ultrasound with an 18 cm 3.5 Mhz linear array transducer (ASP-18).	Beef cattle	Pena et al. [24]
Subcutaneous and abdominal adipose tissue depots.	Scanned prior to slaughter.	At 16 sites on the body surface of the animals.	Toshiba SSA-370A PowerVision 6000 ultrasound machine equipped with a 6 to 12 MHz (PLN-805AT) linear probe and a 2 to 7 MHz (PVM-375AT; all from Toshiba Medical Systems, Neuss, Germany) convex array transducer was used for transcutaneous ultrasonography.	Dairy cattle	Raschka et al. [28]
Loin area, fat thickness and loin depth.	Scanned 24 h before slaughter.	Between the 12th and 13th ribs.	Pie Medical Falco 100, B mode, real-time ultrasound machine with a 6 cm length and an 8 MHz linear probe (The Netherlands).	Lamb	Sahin et al. [29]
Muscle depth and fat thickness.	Scanned before slaughter.	Sternum region at the 3rd and 4th sternebrae. Lumbar vertebra at 2 cm, 4 cm and one-third from the dorsal midline.	Toshiba Sonolayer ultrasound (SAC-32B, Toshiba Corp., Otawara, Japan) with a 5 MHz probe (veterinary model).	Goat	Teixeira et al. [23]
Loin area, total backfat thickness and thickness of the fat layers.	Scanned 24 h before slaughter.	Between the 10th and 11th ribs (10th intercostal space). Behind the last rib (the 14th rib).	Aloka 500 machine (Aloka Holding–Europe, Zug, Switzerland) and a 3.5 MHz, 12 cm long probe (Aloka Holding–Europe, Switzerland).	Pig	Ayuso et al. [30]
Reproductive physiology					
Ovarian activity: corpus luteum rate and score.	Scanned at ~600 days of age.		Honda HS-2000 V or HS-2100 V (Honda Electronics Co., Ltd., Toyohashi, Japan) using a 10 MHz linear array transducer.	Cattle	Corbet et al. [31]
Pregnancy rate and weeks pregnant.	Scanned ~5 weeks after completion of mating in heifers and first-lactation cows.		Honda HS-2000 V or HS-2100 V (Honda Electronics Co., Ltd., Toyohashi, Japan) using a 10 MHz linear array transducer.	Cattle	Corbet et al. [31]
Health of testicles and epididymides.	Scanned when the rams were 13 months old (or older), inside and outside the reproductive season.	Caudal surface of the testicle along its longitudinal axis. Head and tail of the epididymis and the pampiniform plexus.	Ultrasound scanner AMI B7, Alliance Medical, QC, Canada using a 6.0 MHz frequency sector transducer.	Sheep	Gouletsou et al. [32]

Body weight, body fat composition, skin thickness and time of measurement are variables that need to be considered to estimate quantitatively and qualitatively the reliability of ultrasound images in predicting muscle glycogen content in ruminants.

## 4. Estimation of Glycogen Content in Muscle Tissue Using Ultrasound and Other Technologies

The estimation of glycogen concentration in muscles can be challenging but various methods have been developed (Table 2) due to its critical importance, particularly in beef cattle and athletes. There are three methods to detect glycogen in muscle in athletes. The first one is a biopsy, which is undesirable for use in beef cattle as it would cause muscle injury near the time of slaughter which is clearly undesirable. Moreover, the muscle sample would still require laboratory preparation and analysis to estimate the glycogen content.

Nuclear magnetic spectroscopy provides opportunities to determine metabolite changes in fresh meat [33]. However, while magnetic resonance spectroscopy can be used in various species in vivo, a bore large enough for cattle does not yet exist [34], with the largest available bore size being 80 cm. However, even open MR scanners are not practical for commercial use on live cattle, as it would require anesthesia of the animals to acquire the data, making the procedure time-consuming and expensive. Low-field scanners are, at this stage, not capable of providing sufficient field strength to allow for differentiation in the MRS signal. While there are standing magnetic resonance scanners for horses, it is likely these have insufficient field strength to perform magnetic resonance spectroscopy as they would not be able to differentiate peaks of the various metabolites.

Ultrasounds are useful to investigate structures but less so for the quantitative assessments of tissue properties compared to magnetic resonance imaging. Some implications for the use of ultrasounds to measure muscle water/muscle glycogen have been raised.

Muscle water is impacted by multiple factors like lactate concentration, pH, intramuscular fat and hydration levels might have a much higher influence on water levels of the tissue, therefore making the method unreliable in an uncontrolled sample group to estimate glycogen concentration.

Higher glycogen concentrations can be linked to higher water concentrations in muscle tissue [8]. Ultrasounds, therefore, can measure glycogen through the change in echo intensity. However, since other factors might also influence echo intensity, the use of ultrasounds to detect water and glycogen content of the muscle would require a carefully calibrated study. The lack of such validation is likely cause for inconsistent results in an uncontrolled context [35].

Additionally, the development of an accurate ultrasound system to predict glycogen content in production animals is still lacking. Hill and Millan [9] found a high (r > 0.90) correlation between muscle biopsy glycogen and high-frequency ultrasounds in humans. However, human studies are usually undertaken with athletes who typically have low and similar body fat percentages. Similarly, Sarvazyan et al. [36] reported that the prediction of muscle hydration status using an ultrasound velocity might change if the level of dehydration increases. Thus, accurate ultrasound prediction is dependent upon the range of fat, dehydration level, skin thickness and other significant individual variations. High-frequency ultrasounds, which are required for glycogen detection, lack penetration depth, as the higher frequency is linked to a shorter wavelength and increased wave attenuation [37].

Another method that provides real-time and non-invasive measures is microwave sensor technology [35]. In humans, this technology has been studied to detect muscle glycogen, blood lactate, blood oxygenation, water content, and muscle proteins consecutive to exercise-induced muscle damage. Similarly to ultrasound, this technique sends electromagnetic waves of 300 MHz to 300 GHz through the skin and senses reflection, using extremely low levels of radiation. The sensor is based on conductivity (the ability of a material to conduct an electric current) and permittivity (which depends on the dielectric constant of the material). While there have been successful tests to detect glucose and lactate levels, the detection of glycogen was only postulated in Greene et al. [35] but could be associated with other measurable parameters. More recently, ultra-wide-band microwave systems have been applied to determine fat carcass traits in beef cattle with similar precision and accuracy (however, at a faster capturing measurement time) than ultrasound technology [38]. Advances have been made in determining fatness in live cattle [38], beef carcasses [39] and sheep carcasses [40,41,42], with significant applicability to the livestock industry. These developments enable more accurate grading, better management of livestock production and improved meat quality assessment, ultimately enhancing efficiency and profitability within the industry.

Digital infrared thermal imaging might give some insight into body temperature, and in sports medicine, thermal imaging has been postulated to diagnose micro-muscle rupture with increased glycogen usage [43,44]. While this study did not compare glycogen content but rather temperature changes in training, it could be a cost-effective model, if successful. However, there are a lot of factors that have to be considered with thermal imaging, like outside temperature, coat length and drafts.

As an alternative, bioimpedance or bioelectrical impedance analysis [8], if applied at a swept frequency, would provide information about fat, muscle content and potentially water. While controversial in the past, this improved technology with different frequencies is used in the space station to check on muscle and muscle wasting. However, it has to be applied in a standardized manner, and it would have to be used at a swept frequency to provide a spectrum of impedance.

## 5. Estimation of Intramuscular Fat Using Ultrasound and Other Technologies

The development of technologies such as spectroscopies [45], computer image analysis [46] and ultrasound imaging [47] to predict intramuscular fat content has been well appreciated by genetic evaluation programs.

Ultrasound technology has the advantage of being portable, real time, reasonably priced and non-invasive to predict intramuscular fat content. However, ultrasound technologies demonstrated a less accurate anatomical resolution, and the image analyses are not easily automated when compared to other non-invasive methods (e.g., magnetic resonance imaging and computed tomography) [48].

The prediction of intramuscular fat using ultrasound is also affected by breed and gender as reported by Reverter et al. [11] (Table 3). Variation in the prediction of intramuscular fat is observed due to differences in real-time ultrasound software systems, and it can range from a very weak correlation (0.2) up to 0.75 depending on the prediction system and predictor intramuscular fat measurement [49]. Intramuscular fat predictions are often affected by software and the ultrasound operator. In addition, real-time ultrasound systems are less precise in a high marbling score class compared to a low marbling score class [49]. Similarly, when cows were selected with body condition scores from 4 to 7, the animal effect decreased from 9% to 4.9% for % intramuscular fat measured by ultrasound technology [12].

Nunes et al. [47] reported that considerable progress has been made in developing new algorithms to measure rib eye area, back fat thickness and intramuscular fat using ultrasound and color images. Over time, ultrasounds and software configurations have been used to investigate how to best predict fat carcass traits in beef cattle [38,50,51,52,53,54,55]; however, it has not been widely used in cattle, neither by the private sector nor by commercial firms.

The fat content of production animals has been accurately estimated by image analysis techniques such as hyperspectral imaging and computerized image analysis. Hyperspectral imaging consists of spectroscopy, a light source, an area detector and a computer control system to acquire and calibrate the hyperspectral images and parameters of area scanning [56]. The marbling [57] and intramuscular fat concentration [58] of beef can also be estimated at a level of prediction greater than 0.90 by using hyperspectral imaging. However, it has to be taken into account that hyperspectral imaging is not a real-time application as a prolonged time period is required for data acquisition and processing [59].

Computer vision systems were used by Jackman et al. [60] to predict the marbling of raw beef samples. The accuracy of marbling prediction using images was very strong (r > 0.90). However, the authors are aware that this method requires several tests to identify and isolate the region of interest with the image. It can be very challenging to match the measurements of expert graders with the computer system and manual adjustment is sometimes required. Pena et al. [61] report that computerized image analysis is a good technique to predict marbling, but intramuscular fat may be overestimated. In addition, they observed that staining improves the computerized image analysis’ prediction of marbling compared to fresh beef, but since it is time-consuming, it is not easily adapted to the modern meat industry.

While hyperspectral imaging and computerized image technologies can predict fat content with very high accuracy, they were developed for post-slaughter measurements. Ultrasonic techniques were developed over the past decades to accurately predict body composition of live animals, but they lack the accuracy of hyperspectral imaging and computerized image analysis technologies to determine marbling prediction.

More recently, other handheld technologies have been applied to provide intramuscular fat information under commercial conditions. Near-infrared spectroscopy and camera systems are under validation to accurately determine intramuscular fat in lamb (moderate precision R^2^ = 0.40–0.64, RMSEP = 0.70–1.22%) [62] and beef industries (62.6% carcass with accurate prediction) [63], respectively.

## 6. Conclusions/Recommendations

The determination of muscle glycogen in live animals can help farmers manage stress and feeding practices to deliver high-quality, safe and nutritious meat products that meet consumer demands. However, ultrasound technology is not yet a practical method to estimate muscle glycogen levels in cattle; it is relevant to consider standardization challenges due to variations in fat and water content between individuals.

Bioimpedance using multiple frequencies appears to be an alternative to estimating fat mass in humans. A proof-of-concept study would need to be investigated for animals, but it might be a useful tool to assess fat mass in live animals but not muscle glycogen concentration.

Currently, low-frequency ultrasound systems are used to predict intramuscular fat/marbling but there is variation in accuracy depending on intramuscular fat content, breed and the magnitude of the difference between the minimum and maximum values of intramuscular fat in a given population [64]. Alternatively, another ultrasound frequency could be tested to improve the accuracy of intramuscular fat of beef and lamb by increasing the penetration. It will benefit animal genetic programs in the prediction of estimated breeding values (EBVs) and Australian sheep breeding values (ASBVs) to select for animals when they are alive and the industry by updating the value/pricing of carcasses, especially for animals with greater intramuscular fat content.

The advancement of imaging technologies is improving the ability to predict fat content, and hopefully, in the future, these innovations will also enable the prediction of traits that help prevent dark cutting in cattle. This could reduce losses to the industry while enhancing meat quality for consumers.

## Figures and Tables

**Table 2:** Merits and challenges of different methodologies to detect glycogen in muscle.

Method	Merits	Challenges
Biopsy	- Direct and accurate measurement of glycogen content. - Established and reliable technique for glycogen analysis.	- Invasive and undesirable for use in beef cattle due to muscle injury risk near slaughter. - Requires laboratory preparation and analysis.
Magnetic Resonance Spectroscopy (MRS)	- Non-invasive and offers insights into metabolite changes in tissues.	- Not practical for commercial use on live cattle due to high cost and time requirements.
Ultrasound	- Non-invasive and can be used in the field. - Can investigate muscle structures and offer the potential for glycogen content estimation. - Widely available and relatively low cost in comparison to MRI.	- Muscle water can affect the reliability of measurements. - Accuracy depends on individual variations, such as fat levels, dehydration, skin thickness and operator.
Microwave Sensor Technology	- Real-time, non-invasive, and can detect various biomarkers like glycogen, lactate, and muscle proteins.	- Technology primarily used in humans, and its application in cattle is still under development.
Digital Infrared Thermal Imaging	- Non-invasive and could be a cost-effective alternative for monitoring changes in body temperature.	- Cannot directly measure glycogen content. - External factors (temperature, coat length and drafts) can affect accuracy.
Bioimpedance Analysis	- Non-invasive and can provide information on fat and muscle content and possibly water.	- Requires careful calibration and standardized application for reliability.

**Table 3:** Ultrasound, marbling and intramuscular fat correlation in beef cattle.

Cattle Background	US	Ultrasound Prediction and Phenotypic Measure	Accuracy	Reference
Cross-breed steers (mix of Angus, Simmental, Red Angus, Brahman and Hereford).	AUS1 and CVIS: Aloka 500 V system equipped with a 3.5 MHz, 17 cm transducer (distributed by Aloka USA, Inc., Wallingford, CT, USA). AUS2 and PIE: Pie Scanner 200 system equipped with a 3.5 MHz, 18 cm transducer (distributed by Classic Ultrasound Equipment, Tequesta, FL, USA). CPEC1-4: Aloka 210 system equipped with a 3.5-MHz, 12.5 cm transducer (distributed by Aloka USA, Inc., Wallingford, CT, USA).	Ultrasound to predict marbling and marbling score. Ultrasound to predict intramuscular fat and marbling score.	Correlation from 0.63 to 0.75 using CPEC software system. Correlation from 0.22 to 0.30 using AUS software system, 0.74 using CVIS and 0.39 using PIE.	Herring et al. [49]
Angus and Hereford steers and heifers obtained from abattoirs.	Aloka 500 V scanning machines using a 3.5 MHz, 17.2 cm linear array transducer and stored on portable PC using ImageNation frame grabber cards.	Ultrasound to predict intramuscular fat and intramuscular fat measured with near-infrared spectroscopy (NIRS): Angus Hereford	Using the Iowa State University Research Foundation (ISURF) ultrasound software (USOFT/ISURF 01480, 1996) provided by Iowa State University Research Foundation (ISURF) (Ames, IA, USA) and required the placement of an 84 × 84 pixel square box into the image and a subjective grading of the quality of the image. Genetic correlation (SE) of 0.47 (0.21) for bulls and 0.46 (0.15) for heifers. Genetic correlation (SE) of 0.28 (0.27) for bulls and 0.93 (0.11) for heifers.	Reverter et al. [11]
Beef cows of variable breed composition, (primarily *Bos taurus*) ranging from 2 to 13 years old.	Aloka SSD-500 machine (Corometrics Medical Systems, Wallingford, CT, USA) fitted with a 17 cm, 3.5 mHz linear transducer.	Ultrasound to predict intramuscular fat and carcass marbling score.	Residual correlation 0.73 using Scanning Partner software (UltraInsights, Inc., Maryville, MO, USA).	Emenheiser et al. [12]

## Data Availability

No new data were created or analyzed in this study. Data sharing is not applicable to this article.
